# P161: Knowledge and perception toward hand hygiene among health- care workers in teaching hospital, Korea

**DOI:** 10.1186/2047-2994-2-S1-P161

**Published:** 2013-06-20

**Authors:** EK Kim, MK Joo, SY Baik, SK Hong

**Affiliations:** 1 Infection Control Office, CHA Bundang Medical Center, CHA University, Soengnam-si, Gyeonggi-do, Korea, Republic Of; 2 Department of internal medicine, CHA Bundang Medical Center, CHA University, Soengnam-si, Gyeonggi-do, Korea, Republic Of

## Introduction

Hand hygiene is considered the most important infection control measure in health care setting and forms the core of patient safety. Despite the activity of hand hygiene promotion continued, we observed that hand hygiene compliance is congested. This study was performed in order to survey the knowledge and perception toward hand hygiene for health-care workers(HCWs) to utilize further activity of hand hygiene promotion.

## Methods

This study was performed between January and March 2013 in CHA Bundang medical center, 865-beds teaching hospital in Korea. Our survey material used the WHO questionnaire revised August, 2009. The questionnaire included 7 questions on general characteristics, 10 on knowledge issues(25 scoring), 11 on perception issues(96 scoring). The collected data were analyzed using the SPSS(ver. 20.0) program.

## Results

During the study period, 348 HCWs were surveyed. The surveyed HCWs were nurses(55.5%), physicians(4%), technicians(25.6%), and nurse assistants(14.7%). The mean age was 30.2(*SD*=6.6) and the majority of participants were female(76.4%). The mean score of hand hygiene knowledge was 14.25(*SD*=2.05), there were significantly differences in gender (female, *t*=-2.276, *P*=.023), clinical experience(above 5years, *t*=-2.463, *P*=.014), profession(nurse, F=9.337, *P*<.01) and no significantly differences in age, department. Otherwise the mean score of hand hygiene perception was 75.2(*SD*=11.83), there were significantly differences in age(above 31years, *t*=-3.224, *P*=.001), profession(nurse, F=4.1, *P*=.007), department(ICU, F=2.57, *P*=.038) and no significantly difference in gender, clinical experience. Having had a formal training in hand hygiene was significantly difference both of knowledge(*t*=5.50, *P*<.01) and perception(*t*=2.4, *P*=.017).

## Conclusion

In this study, the knowledge and perception of hand hygiene for HCWs is low, and It could result in congesting hand hygiene compliance. Therefore, it is necessary to develop a promotion program to build knowledge and perception of hand hygiene to improve compliance for HCWs.

## Disclosure of interest

None declared

